# Urinary Metabolic Distinction of Niemann–Pick Class 1 Disease through the Use of Subgroup Discovery

**DOI:** 10.3390/metabo13101079

**Published:** 2023-10-13

**Authors:** Cristóbal J. Carmona, Manuel German-Morales, David Elizondo, Victor Ruiz-Rodado, Martin Grootveld

**Affiliations:** 1 Andalusian Research Institute on Data Science and Computational Intelligence, University of Jaen, 23071 Jaen, Spain; ccarmona@ujaen.es (C.J.C.); mgerman@ujaen.es (M.G.-M.); 2 Leicester School of Pharmacy, De Montfort University, The Gateway, Leicester LE1 9BH, UK; 3 School of Computer Science and Informatics, De Montfort University, The Gateway, Leicester LE1 9BH, UK; elizondo@dmu.ac.uk; 4 Pivotal Contract Research Organisation, Community of Madrid, Calle Gobelas 19, La Florida, 28023 Madrid, Spain; vruizrodado@gmail.com

**Keywords:** Niemann–Pick disease type C1 (NPC1) disease, NMR analysis, NMR-linked metabolomics, chemical pathology, imbalanced data, subgroup discovery, evolutionary fuzzy systems

## Abstract

In this investigation, we outline the applications of a data mining technique known as Subgroup Discovery (SD) to the analysis of a sample size-limited metabolomics-based dataset. The SD technique utilized a supervised learning strategy, which lies midway between classificational and descriptive criteria, in which given the descriptive property of a dataset (i.e., the response target variable of interest), the primary objective was to discover subgroups with behaviours that are distinguishable from those of the complete set (albeit with a differential statistical distribution). These approaches have, for the first time, been successfully employed for the analysis of aromatic metabolite patterns within an NMR-based urinary dataset collected from a small cohort of patients with the lysosomal storage disorder Niemann–Pick class 1 (NPC1) disease (*n* = 12) and utilized to distinguish these from a larger number of heterozygous (parental) control participants. These subgroup discovery strategies discovered two different NPC1 disease-specific metabolically sequential rules which permitted the reliable identification of NPC1 patients; the first of these involved ‘normal’ (intermediate) urinary concentrations of xanthurenate, 4-aminobenzoate, hippurate and quinaldate, and disease-downregulated levels of nicotinate and trigonelline, whereas the second comprised ‘normal’ 4-aminobenzoate, indoxyl sulphate, hippurate, 3-methylhistidine and quinaldate concentrations, and again downregulated nicotinate and trigonelline levels. Correspondingly, a series of five subgroup rules were generated for the heterozygous carrier control group, and ‘biomarkers’ featured in these included low histidine, 1-methylnicotinamide and 4-aminobenzoate concentrations, together with ‘normal’ levels of hippurate, hypoxanthine, quinolinate and hypoxanthine. These significant disease group-specific rules were consistent with imbalances in the combined tryptophan–nicotinamide, tryptophan, kynurenine and tyrosine metabolic pathways, along with dysregulations in those featuring histidine, 3-methylhistidine and 4-hydroxybenzoate. In principle, the novel subgroup discovery approach employed here should also be readily applicable to solving metabolomics-type problems of this nature which feature rare disease classification groupings with only limited patient participant and sample sizes available.

## 1. Introduction

Niemann–Pick type C disease (NP-C, OMIM 257220) is a neurodegenerative lysosomal storage disease that arises from defects in either the type C1 or C2 genes [1] and involves the storage and modified cellular trafficking of cholesterol and sphingolipids, together with diminished acidic store calcium levels. It typically presents in childhood with dementia, vertical ophthalmoplegia, dystonia, hepatosplenomegaly and ataxia. Moreover, some early-onset cases are fatal in view of severe neonatal liver disease. Adult onset also occurs, and this form of the debilitating condition is typically associated with neuropsychiatric presentation [1]. Currently, diagnosis continues to be largely based on clinical presentation, the application of techniques for filipin staining of skin fibroblasts and sequencing of the gene(s) to identify the mutations [1] (filipin staining remains an acceptable tool for the identification of intracellular cholesterol deposits). Moreover, neuroimaging of patients afflicted with this neurodegenerative disease can reveal delayed myelination or parietal white matter changes, and also a thinning of the corpus callosum, in addition to supra- and infratentorial atrophies [2,3]. Niemann–Pick type C disease’s pathological cascade involves neuroinflammation, neuronal apoptosis and oxidative stress, phenomena which are all probable contributory factors towards the clinical phenotype [4]. 

Currently, biofluid biomarkers available for NPC1 disease and its prognosis are somewhat limited, although recent developments have gone some way in establishing that the determination of selected biomolecules in blood samples collected from these patients may offer at least some valuable advantages for diagnosing and perhaps monitoring the status of this disorder. Indeed, recently, NPC1 disease diagnosis has been markedly promoted via the uncovering of reliable biomarkers of high specificity and sensitivity that are upregulated in blood samples collected from patients with this disorder. These important developments are extensively reviewed in ref. [5], the most encouraging including selected oxysterols [6], lysosphingomyelin [7], *N*-(3β,5α,6β-trihydroxy-cholan-24-oyl)glycine (known as bile acid B) [8] and a previously unknown lipid (lysoSM-509), which has now been characterised and identified as *N*-palmitoyl-*O*-phosphocholineserine, the most abundant species of the *N*-acyl-*O*-phosphocholineserine lipid classification [9]. Globally, the oxysterol cholestane-3β,5α,6β-triol is now the most widely accepted laboratory-determinable biomarker for NPC1 disease and has become a favoured first-line test for its diagnosis, along with genetic analyses [9]. However, interestingly, lysoSM-509 has exhibited a strikingly similar diagnostic potential to this oxysterol [9]. Further valuable biomarkers available for this lysosomal storage disease include those arising from proteomic analysis [10]. 

Despite the availability of much supporting information regarding the clinical aspects and development of NPC1 disease [1], issues regarding its reliable diagnosis, laboratory-based or otherwise, still persist, as indeed they do for strategies involving the tracking of its progression, in both untreated patients and those receiving approved or experimental therapies. For example, high concentrations of the oxysterol cholestane-3β,5α,6β-triol, together with that of 7-ketocholesterol, have been found in the blood plasma of patients with neonatal cholestasis as well as those with NPC1 disease, and this represents a significant precaution of the use of these agents as molecular markers for the latter [11]. Moreover, some significant overlap between the *N*-palmitoyl-*O*-phosphocholineserine levels of NPC1 and newborn healthy control patients severely restricts its utility as an NPC disease biomarker for screens of dried blood spot samples collected from newborns [9]. Additionally, detailed information available regarding the cellular mechanisms giving rise to neuronal cell death remains limited to date. Therefore, further information is required in order to allow us to develop multiple points of intervention in this disease process that could supplement existing therapies, and the virtually noninvasive, multi-analyte monitoring of virtually intact biofluid samples by high-resolution proton (^1^H) NMR analysis, coupled with linked multivariate (MV) metabolomics analysis, may serve to provide valuable diagnostic and prognostic outputs for NPC1 disease, most notably because of its ability to rapidly provide datasets often containing >100 detectable metabolites and their concentrations per sample. Hence, multi-analytical ^1^H NMR-based metabolomics analysis of both biofluids and tissues offers a high level of potential regarding the investigation of metabolic processes and serves as an extremely powerful means of probing, for example, the biochemical basis of human disease aetiology. Indeed, this form of combined analysis has been extensively employed in a very wide range of biomedical and clinical investigations, including the identification of diagnostic biomarkers, e.g., refs. [12,13,14].

However, for lysosomal storage diseases such as NPC1, such studies are hampered by the very limited availability of biofluid samples for their investigation. Therefore, in this investigation, we employed a global multicomponent high-resolution proton ^1^H nuclear magnetic resonance (NMR) analysis approach coupled with a newly developed computational intelligence technique (CIT) in order to distinguish the urinary ^1^H NMR profiles of NPC1 patients from those of their corresponding heterozygous carrier controls. This work was performed to allow us to explore the nature of metabolic disturbances in NPC1 disease-active patients which are represented as unusual biomolecular ‘signatures’ detectable within the ^1^H NMR profiles of biofluid samples. Indeed, this strategy is highly suited to the provision of effective and reliable MV predictions when the number of potential explanatory (X) variables available (P) exceeds the sample size (n), a problem commonly encountered in experimental practice in view of the multidimensional nature of such ‘omics’-type investigations, and the restricted numbers of samples available from patients with rare metabolic and/or neurodegenerative diseases.

The complexity of the problem leads to the necessity of using methodologies with the ability to build models and analyse MV datasets. There is a methodology known as knowledge discovery in databases [1], which is widely used throughout the literature; these are also known as data mining processes. Data mining is best suited to current data environments in ‘real world’ problems, such as abnormal respiratory event detection in sleep [15], or coronary heart disease [16], since nowadays data volumes are markedly expanding [17], and hence data contents are becoming increasingly complex, and problems are changing more rapidly than those previously encountered.

For this contribution, the application of a technique within the data mining descriptor known as Subgroup Discovery [18,19] was applied. This approach serves as a descriptive data mining technique using supervised learning. It is placed midway between classification and description, where given a descriptive property of a dataset (the disease class variable of interest, or target variable), the objective is to discover subgroups with behaviours which differ from those of the complete set (that is, with a different statistical distribution). As an example, in cases where a medical institution may wish to gain knowledge regarding the circumstances under which a patient may suffer from cancer, its intention is not to predict cancer, but to understand the risk factors that may lead to this condition and how to avoid it.

The knowledge extracted for SD models is represented in the form of rules, the high interpretability of this type of strategy allowing experts to comprehend why certain decisions or predictions have been made in a facile manner. In machine learning, it is considered that a model is better interpretable than an alternative if its decisions are easier for a human to comprehend than those from the other model. The concept of interpretability is very close to explicability [20]. In fact, we can find interesting applications of SD to “real-world” problems throughout the literature thanks to the implicit interpretability of SD algorithms [21,22]. Amongst the most important aspects of the algorithms within SD [23] is the search strategy, together with relevant associated quality measures. It should also be noted that the quality measures represent key factors in order to guide the search process and to measure the quality of the algorithm employed. The analysis of quality measures in SD is complex, and hence we have presented a complete description of them in Appendix A.

For the analysis of this metabolomics problem, we primarily employed the NMEEFSD algorithm [24], which is one of the most representative algorithms within the SD task. This is a multi-objective evolutionary fuzzy algorithm that obtains a set of general and accurate fuzzy rules from the use of original genetic operators. It combines the properties and advantages of the evolutionary algorithms [25], together with fuzzy logic [26], which is known in the community as evolutionary fuzzy systems [27]. A complete description of this class of systems for SD tasks is provided in Appendix B. It is, however, important to note that the NMEEFSD algorithm employed in this contribution contains a modification of the original one, with a new screening function based on one statistical test which is discussed subsequently.

The study also commences with a predictive analysis in order to show the competitiveness with respect to the accuracy of the SD algorithm. Indeed, for this case study, it is of much importance that extracted knowledge is accurate. A comparison with the most relevant predictive algorithms is performed. Furthermore, in view of the complex nature of the problem presented, it was necessary to apply preprocessing algorithms to the original dataset in order to balance the number of examples collected from patients with NPC1 disease. Indeed, this dataset has a very low number of samples available for exploring metabolic imbalances associated with it, and such preprocessing should improve the results derived from the predictive algorithms employed. Once the precision of the NMEEFSD algorithm in predictive induction had been demonstrated, a descriptive analysis from the SD point of view was performed. This represented the primary major objective of the study. The secondary objectives of this work were to provide valuable urinary molecular information as a valuable aid to the diagnosis of NPC1 disease, with special reference to facilitating our understanding of imbalances in metabolic pathways, which are involved in the pathogenesis of this disease process. These objectives are listed below:

**Objectives of the study**

- **Major Primary Objective: To employ SD technologies for the discovery of NPC1 disease subgroups with behaviours that are differentiable from those of the complete dataset;**
- **Secondary Objective (1): To provide urinary biomarker information, which is valuable for the diagnosis and prospective status monitoring of NPC1 disease;**
- **Secondary Objective (2): To explore these urinary biomarker subgroup discovery patterns in order to preliminarily detect any metabolic pathways that are impaired or disturbed in NPC1 disease (a process that may provide useful chemopathological and drug-targeting information).**

Overall, the classificational success rate of the subgroup discovery technique applied allowed us to successfully distinguish between the NPC1 disease patients and their parental heterozygous controls. Moreover, this analysis provided much useful information regarding profound disease-induced disturbances in metabolic pathways in this condition, most notably the combined tryptophan–nicotinamide, tryptophan, kynurenine and tyrosine metabolic pathways. In principle, up- or downregulated metabolites from these pathways may serve as valuable biomarkers for this and related diseases.

## 2. Materials and Methods

The study presented in this contribution is the result of the combination of different novelty technologies. Firstly, a high-field proton (^1^H) NMR spectroscopic analysis is employed in order to recognise urinary biomolecular signatures which are characteristic of NPC1 disease. This extraction of information is actually very interesting because the use of this methodology is fast, and most especially noninvasive. Moreover, the use of data mining techniques based on rule-based systems shows high-quality results with very interpretable descriptions for this clinical condition.

**Figure 1:**
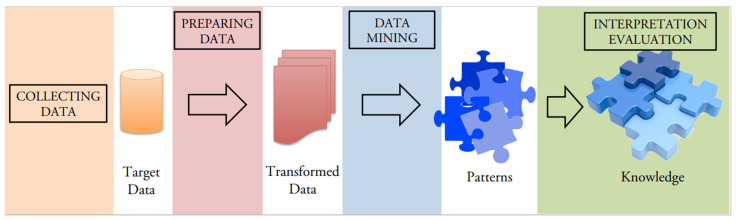
The knowledge discovery in databases process performed in this contribution.

Below is a flowchart of the study presented in the following subsections which is based on the main stages of a Knowledge Data Discovery [28] (Figure 1). The original stage concerning the collection of urine samples and data from their ^1^H NMR analysis is presented in Section 2.1. Next, Section 2.2 and Section 2.3 present the complete process in order to prepare data for further implementation of the data mining stage. Subsequently, Section 2.4 and Section 2.5 outline details regarding the determination of urinary pH values and the effects of adding 10% (*v*/*v*) on this parameter, respectively. Section 2.6 then addresses the data mining phase of this study, along with the validation of these models (Section 2.7), and Section 2.8 provides experimental information on the performance of metabolic over-representation analysis from the key metabolic biomarker data provided. Section 3 then presents the patterns extracted in the problem, and finally, Section 4 reveals the knowledge discovered in the experimental study and discussions considered.

### 2.1. Sample Collection, Preparation and Storage

Urine samples (*n* = 52) were collected from groups of *n* = 12 NPC1 disease patients (untreated) and their corresponding heterozygous (parental) carriers (*n* = 40). These UK-based studies were collected with informed consent and approved by the appropriate Research Ethics Committee (06/MRE02/85).

Urine samples collected from each of these participants were routinely stored at −80 °C, and when ready for analysis, these were thawed and centrifuged to remove cells and debris (5000 rpm for a period of 10 min). Aliquots (0.60 mL) of the supernatants arising therefrom were then thoroughly rotamixed with 0.07 mL of deuterium oxide (^2^H_2_O), and these samples were then directly transferred to 5-mm diameter NMR tubes. Prepared samples that were not immediately analysed by NMR thereafter were stored in a refrigerator at 4 °C for a maximum duration of 24 h prior to analysis.

### 2.2. ^1^H NMR Analysis of Urine Samples

^1^H NMR spectra of these samples were acquired on a Bruker AV-600 spectrometer. Chemical shifts were internally referenced to the -CH_3_ group resonances of acetate (*s*, δ = 1.920 ppm), alanine (*d*, δ = 1.487 ppm), lactate (*d*, δ = 1.330 ppm) and creatinine (>N-CH_3_ *s*, δ = 3.030 ppm). The identities of biomolecule resonances present in the complete urinary ^1^H NMR spectra acquired were routinely assigned by a consideration of chemical shift values, coupling patterns and coupling constants, and then cross-checked with the *Human Metabolome Database* (HMDB). Biofluid dataset matrices were generated through the application of macro procedures for line broadening, zero-filling, Fourier transformation and phase and baseline corrections, followed by the application of a separate macro for the “intelligent bucketing” processing subroutine; all procedures were performed within the ACD/Labs *Spectrus Processor 2019* software package (ACD/Labs, Toronto, ON, Canada M5C 1T4). Before performing this bucketing process, all spectra were visually inspected for any distortions and manually corrected, if required. Spectral regions containing resonances arising from ethanol (δ = 1.20–1.22 (t) and 3.63–3.67 ppm (q)), which were detectable in several of the heterozygous carrier control urine samples, and urea (broad, δ = 5.58–5.98 ppm) were removed from all spectra, in addition to that of the intense H_2_O/HOD signal (δ = 4.65–5.16 ppm).

### 2.3. Data Preprocessing

The experimental strategy employed involved the addition of all spectra acquired into one common file, in which the “intelligent bucketing algorithm” examined all spectra simultaneously and focused on the “bucket limits” of commonly observed resonance intensity areas. This strategy generated one global table of intelligently selected bucket (ISB) intensities which was then imported into MS Excel for further manipulation. For the purposes of multivariate data analysis, all urinary ISB intensities were normalised to the urinary creatinine (Cn) > N-CH_3_ group resonance (δ = 3.03 ppm (*s*)), this signal arising from protons that do not exchange with deuterium from the added ^2^H_2_O field frequency lock. This ISB approach ensured that all bucket edges involved did not coincide with ^1^H NMR resonance maxima, and this process therefore prevented the splitting of signals across separate integral regions; a 0.04 ppm bucket width with a 50% looseness factor was employed (ISB bucket ranges for the above reference acetate-, alanine-, lactate- and creatinine-CH_3_ group resonances were δ = 1.89–1.95, 1.45–1.50, 1.32–1.36 and 3.00–3.06 ppm, respectively). These datasets were then cube-root-transformed and Pareto-scaled prior to performing multivariate data analysis. Subsequently, principal component analysis was employed in order to screen for any outliers (none were detectable in plots of PCs 2-5 vs. 1; data not shown).

In view of the complications provided by datasets containing a very large number of possible predictor variables, together with a small sample size for one of the groups evaluated (the NPC1 disease one), we elected to reduce the number of the variables available. For this purpose, primarily the ^1^H NMR profiles were subdivided into four separate and distinct regions, specifically the high-field (aliphatic) region (0.52–2.94 ppm), the medium-field region (3.06–4.55 ppm), the medium-to-low-field region (4.99–6.71 ppm) and, finally, the low-field region (6.71–9.43 ppm). Preliminary studies were applied to each separate dataset, but from this, only the low-field region, which contains signals from a wide range of aromatic, purine, pyrimidine and nicotinate/nicotinamide metabolites (along with hippurate, indoxyl sulphate and formate), was found to provide excellent discriminatory results, and therefore only this region was employed for further data analysis purposes in the current study. Following further essential editing and noise reduction, this spectral region was reduced to a total of 54 potential predictor ISB variables.

Preliminary principal component analysis (PCA), two-sample *t* tests and metabolite set qualitative over-representation enrichment analysis were performed using *MetaboAnalyst 5.0* software options. Additional univariate statistical tests (two-sample *t* tests and ANOVA) were performed using the *XLSTAT2020* software package (Addinsoft Ltd., Paris, France). 

### 2.4. Urinary pH Values

The pH values of all urine samples utilised for this study prior to their preparation for ^1^H NMR analysis were determined using a precalibrated CE-approved pH meter (Fisher Scientific Ltd., Loughborogh, UK). Mean ± Standard Deviation (STD) pH values for the NPC1 disease group were 6.37 ± 0.67 (*n* = 12, range 4.78–7.41), whereas those for the heterozygous carrier group were 6.27 ± 0.56 (*n* = 40, range 5.22–7.30). These mean values were both within the known healthy adult control reference range for urinary pH [29]. A two-sample *t* test conducted with 10,000 simulations confirmed that there was no significant difference between the mean pH values of these two disease class groups (*p* = 0.85); prior assumptive tests for non-normality and heteroscedasticity of these data were also found not to be statistically significant.

### 2.5. Investigations of the Potential Influence of Added ^2^H_2_O on the pH Values of Urine Samples during Sample Preparation Stages

In order to explore the influence of the addition of a 10% (*v*/*v*) volume of ^2^H_2_O to NMR analyte admixtures on the pH values of freshly collected urine samples, we designed an experiment to assess the nature and extent of any differences observed from this process, and whether or not adding such a small volume of this NMR field frequency lock can combat against the quite powerful buffering capacity of human urine. For this purpose, a total of *n* = 6 healthy control nonsmoking human participants were recruited (4 females/2 males, mean ± STD age 22.67 ± 1.82 years), and each of these donated a single morning collection urine sample following a strict 8 h pre-fasting episode requested on our participant information sheet, with sample collection occurring immediately prior to the consumption of any foods or beverages, or the use of any toothpastes or other oral healthcare products. Collection of urine samples for this part of the investigation was approved by the Faculty of Health and Life Sciences Research Ethics Committee, De Montfort University (Reference number 457249).

**Figure 2:**
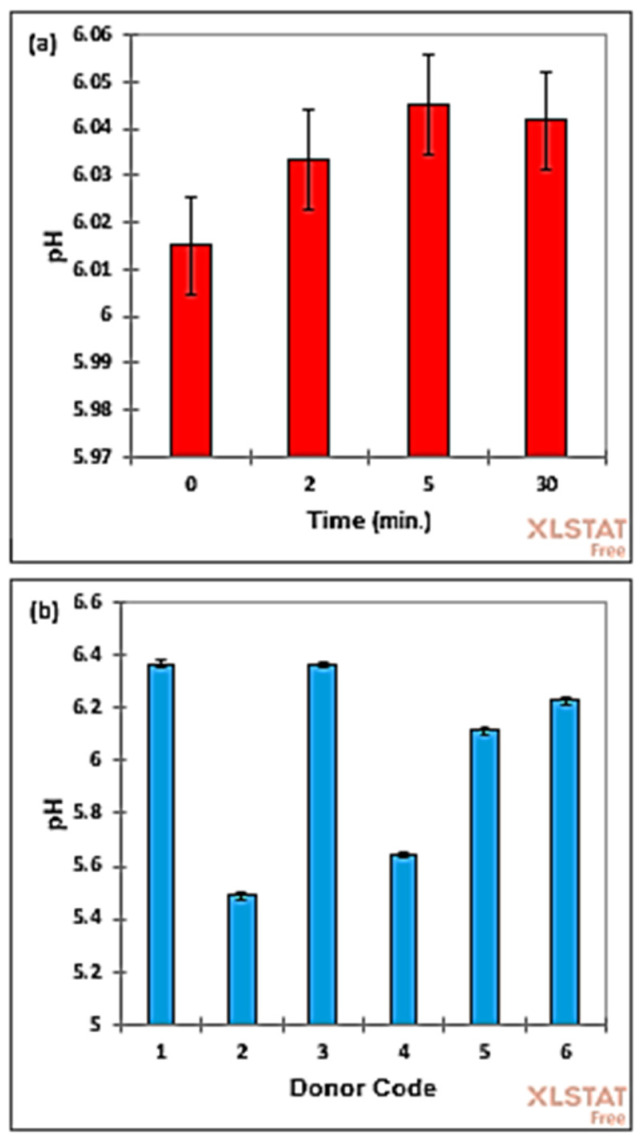
(**a**) Mean (± 95% CIs) for the *n* = 4 sets of urine samples for investigating the influence of added ^2^H_2_O on their pH values. Code 0 represents pH measurements made immediately following collection, and codes 2, 5 and 30 those which were determined at 2.0, 5.0 and 30.0 min following ^2^H_2_O addition as specified in Section 2.5. (**b**) Mean (±95% CIs) for sets of urine samples provided by the *n* = 6 participant donors, and showing ANOVA-determined extremely highly significant differences between them.

A two-factor randomized blocks analysis-of-variance (ANOVA) model was then implemented, and pH measurements were made for each urine sample unequilibrated and equilibrated with 10% (*v*/*v*) ^2^H_2_O (before and at 2.0 min thereafter), and then at 5 and 30 min following donation and ^2^H_2_O treatment, the 30 min sampling time point approximately corresponding to the maximum length of laboratory preparation duration required for ^1^H NMR analysis. Although unexpectedly there were significant differences observed between the mean values for the zero control and 5 and 30 min post-^2^H_2_O addition sample collection/preparation criterion stipulated (*p* = 3.07 × 10^−3^), the very minor, albeit highly reproducible, increases observed in pH value were only 0.50 and 0.45%, respectively (Figure 2a), and this clearly demonstrates that the addition of a 10% (*v*/*v*) content of ^2^H_2_O to urinary NMR samples during their preparation gives rise to only a very negligible pH rise, which will certainly not affect the spectral chemical shift values of ^1^H resonances, nor that of the bucket ranges arising from the computationally intelligent bucketing of these signals. Typically, the mean rise in urinary pH observed 5 min after adding 10% (*v*/*v*) to pre-fixed volume urine samples was only 0.03 (Figure 2a), and this corresponded to a decrease in urinary H^+^ ion concentration of only 7%, which was somewhat lower than that expected from simple water dilution of the sample without allowing for any significant urinary buffering effects.

Mean (95% confidence intervals, abbreviated CIs) pH values of these samples were 6.015 (6.004–6.026) for the zero (untreated) control, and 6.033 (6.023–6.044), 6.045 (6.034–6.056) and 6.042 (6.031–6.052) for the 2, 5 and 30 min post-^2^H_2_O addition samples, respectively. 

Typical mean reference values for concentrations of the most powerful urinary buffering agents available are total phosphate (7.8 mmol/L) [29,30]; total carbonate (none detectable immediately following collection, but up to ca. 50 mmol/L following storage) [28,29]; citrate (males and females, 0.6–4.8 and 1.3–6.0 mmol/L, respectively [31]); and ammonia (simulated ‘free’ level 18 μmol/L, total 28.2 mmol/L [29,30]). During time-dependent urea hydrolysis, however, further NH_3_ is liberated from this source in stored urine. Therefore, it may be concluded that, overall, this powerful buffering capacity of human urine is much more than sufficient to counteract any very minor changes in pH induced by the addition of only 10% (*v*/*v*) ^2^H_2_O.

In 2013, Schreier et al. [32] explored how changes in urinary pH would influence the ^1^H NMR quantification of metabolites in this biofluid. This parameter was varied via the addition of strongly acidic or basis standards to yield values that were within the physiologically observed range, specifically 2.90–9.15 before subsequent artificial buffering and 6.62–7.64 thereafter. From this investigation, the authors concluded that such artificial variation of urinary pH in this manner, even at the extreme values monitored, gave rise to only marginal effects on the quantification of single metabolites, with the exception of urea. Indeed, typically the small changes observed were lower than ±15–20%, a limit which is recommended by the FDA for a mean of 5 repeated determinations when compared to their known theoretical values [33]. For these pH ranges, no pH-dependent effects were noted for the resonances of glucose, lactate, citrate, and phenylacetylglycine, which all remained within the above-15% limit. Moreover, creatinine, which largely remained within these 15% limits, rarely exceeded 20% variation. However, the only metabolite that was found to be influenced by the urinary pH parameter was urea, which showed an almost 50% deviation from its reference level, most notably at the lower pH values investigated.

Furthermore, as expected, no pH value differences whatsoever between ^1^H NMR analysis urine solution supernatants containing ca. 10% (*v*/*v*) ^2^H_2_O were evident from the *ACD/Labs Spectrus Processor* intelligent bucketing of resonances within the low-field region selected for this study (δ = 6.71–9.43 ppm), with all ISBs selected and optimized predominantly leading to the integration of interference-free signals, i.e., those assignable to one or two proton-intense signals arising from a single urinary metabolite only. 

Moreover, as anticipated, differences between the mean pH values of the *n* = 6 participants included in this phase of the study were indeed very highly significant (*p* = 9.42 × 10^−23^), with ranges in their mean values being 5.49–6.37. However, these values were within those reported for healthy adult human participants of 6.0–7.5, with those lying within the 4.5–6.0 and 7.5–8.0 ranges also not representing any major cause for concern [34].

### 2.6. Analysing Data

Once data were collected, prepared and optimized, the main stage of the knowledge discovery in databases was carried out, i.e., the data mining process. Specifically, this process is pe formed and analysed from different viewpoints, both predictive and descriptive analysis. All algorithms used in this contribution are implemented in the KEEL tool-kit (https://www.keel.es (accessed on 10 June 2023)) [35].

#### 2.6.1. Predictive Analysis

One of the complications that renders the extraction of useful information in datasets difficult (as in the present work) is the problem of classification with imbalanced data [36,37]. As noted above, the dataset is imbalanced in view of the number of samples collected from the heterozygous carrier classification being greater than those with NPC1 disease, specifically the imbalance ratio is equal to 3.33. Therefore, the low number of examples with NPC1 disease (only 12) gave rise to major difficulties regarding effective CIT analysis.

This experimental study with the most representative data mining algorithms within predictive induction was performed in the following manner:

- The C4.5 algorithm [38] is the most known throughout the scientific literature and represents a decision-tree-generating algorithm that induces classification rules in this form from a set of given examples. C4.5 is based on the ID3 algorithm, and the main objective is to determine a decision tree that, on the basis of answers to questions regarding the input attributes, correctly predicts the identity or value of the target attribute.
- The FURIA algorithm [39] is a fuzzy rule learner based on the RIPPER implementation. FURIA does not use default rules, and it has special pruning procedures with respect to RIPPER. Its major objective is to extract a compact set of effective fuzzy rules from numerical data, and it has been shown to exhibit excellent behaviour in real-world problems with the same characteristics as the dataset analysed here.
- The k-NN algorithm [40] is the standard classification algorithm based on instances. The class of a given instance is assigned as the majority class with respect to its K closest instances according to a distance measure. The functioning of this algorithm is facile where, for example, for it to be classified, the K-nearest-neighbours method is applied. In this manner, the class proposed for the instance is the majority class in the very next vicinity of the instances where the vicinity is defined as the K instances with a lower distance for the instance to classify.
- The SMO [41] is a sequential minimal optimisation algorithm for training a support vector classifier, and its main objective is to build a support vector machine model with the training set, which then classifies all test data by means of the trained model using the SMO procedure.

To confront the imbalanced nature of collected data, different strategies developed at both data and algorithmic levels have been proposed throughout the literature. Indeed, the goal of solutions at the data level is to obtain a more balanced class distribution dataset that allows a standard classifier to perform in a similar manner, as in a balanced scenario. In this contribution, we have employed oversampling methods [42] that create a superset of the original dataset by creating new instances from minor-class ones. In this manner, the predictive analysis has been performed with the original dataset and with the application of a preprocessing in the original dataset with the SafeLevelSMOTE algorithm [43] in order to balance it.

#### 2.6.2. Descriptive Analysis

The descriptive analysis was carried out with the NMEEFSD algorithm [24]. This is a multi-objective evolutionary algorithm for SD. Its main objective is the acquisition of a set of general and accurate fuzzy rules from the use of diverse genetic operators and a screening function based on the confidence. However, for this contribution, the screening function has been replaced by Fisher’s exact test [44,45], where only rules with a significance level α below 0.10 are considered, i.e., a rule with a value below α = 0.10 rejects the null hypothesis, so this rule is interesting because there are significant differences between the examples of proportions covered. This statistical test is an exact one, since the significance of the deviation from the null hypothesis can be calculated exactly rather than relying on an approximation that becomes exact in the limit as the sample size grows to infinity. The value of this test (*TEF*) is incorporated in the descriptive experimental study.

The knowledge extracted involves the rules [46]:

*R*: *Cond* → *Target*_*value*_

where *Cond* is a set of pairs attribute-value, and *Target_value_* is the value of the class analysed for the algorithm.

The most relevant analyses from the SD point of view [19] are:

- *Interpretability*. A SD proposal must obtain few rules containing a low number of variables in the antecedent part in order to help researchers to understand and use the extracted knowledge, i.e., simple and interpretable subgroups are preferred in the SD task.
- *Trade-off sensitivity and confidence*. These quality measures are relevant in SD because they indicate the percentage of positive examples covered, with the highest possible precision, respectively.
- *Interest*. Rules must provide unusual and interesting information within datasets. This objective is solved through the *unusualness* quality measure because it contributes to interest, generality and confidence in the problem.

As noted above, all of these concepts are represented through quality measures presented in Appendix A.

### 2.7. Validation

Validation of the predictive models must be performed through the use of quality measures based on a confusion matrix (Table 1), which records correctly and incorrectly recognized examples for each class. The most used empirical measure, accuracy (Equation (1)), is not able to distinguish between the number of correct labels of different classes. Specifically, in imbalanced problems, it may lead to erroneous conclusions.

(1) $$Accuracy=\frac{TP+TN}{TP+FN+FP+TN}$$

One appropriate metric that can be employed to measure the performance of classification over imbalanced datasets is the Receiver Operating Characteristic (ROC) graphics [47]. In these graphics, the trade-off between the benefits and costs can be visualized. Indeed, they show that any classifier cannot increase the number of true positives without also increasing the false-positive rate. The Area Under the ROC Curve (AUROC) [48] corresponds to the probability of correctly identifying which of the two stimuli is noise and which is signal plus noise. AUROC provides a single number summary for the performance of learning algorithms.

Computation of the AUROC value is completed through determining the area using the formula of Equation (2), where TP_rate_ is the ratio of examples of the positive class that are well classified, and FP_rate_ is the ratio of examples of the negative class misclassified.

(2) $$AUROC=\frac{1+{TP}_{rate}-{FP}_{rate}}{2}$$

These experiments are considered with a 5-fold stratified cross-validation model; for example, 5 random partitions of data with a 20% maintenance of a priori probabilities of each class, and the combination of 4 of them (80%) as training and the remaining ones representing a test set. Finally, validation of the results obtained for the SD algorithm is considered with the whole dataset, and the quality measures involved are presented in Appendix A.

### 2.8. Qualitative Over-Representation Network Enrichment Analysis (ORA)

For the ORA conducted in this study, which was performed using the *MetaboAnalyst 5.0* software Enrichment Analysis option, lists of metabolite names were entered as a single-column dataset for the single biofluid involved (human urine). Following standardisation of compound labels, the *Metabolic-Pathway*-associated metabolite sets, which contained 99 set entries, were chosen. Enrichment ratios for each pathway were computed by the number of positive metabolite ‘hits’ divided by that expected from the number of all possible metabolites and pathways available in the set.

Three different classes of Subgroup-discovered biomarkers were developed as models for this analysis: (1) only metabolites featured in NPC1 disease-distinguishing rules R6 and R7 were featured; (2) for all rules developed (Rules 1 to 7), all biomolecules which deviated from their study-defined “normal” creatinine-normalised urinary concentrations were included; and (3) all metabolites implicated in all 7 rules as directive SD variables were incorporated in the ORA enrichment analysis model applied.

## 3. Results

### 3.1. AUROC Results

Results obtained in AUROC for the different algorithms are presented in Table 2. As observed, there are two different values. Firstly, the AUROC without preprocessing (*Original*) is presented, and the results obtained with previous application of the SafeLevelSMOTE (*S LS MOTE*) preprocessing method to the original dataset are shown. In addition, the best average AUROC results for each experimental study with and without preprocessing are highlighted.

For the first problem (first column), the benefit obtained with the C4.5 algorithm is clear. This is particularly reflected by the AUROC value with substantial differences with respect to the remaining algorithms. In fact, the difference between the values obtained for C4.5 and the remaining models is relatively high (differences between 7 and 10%). As noted, this algorithm represents the information in a decision tree, and in this manner, the knowledge extracted is very representative of evaluators. Therefore, researchers could analyse this knowledge in rules in order to represent the trees obtained in an easy manner, and they could then analyse and provide essential clues toward our understanding of the metabolic basis of NPC1 disease in this problem.

However, in the results obtained with the preprocessing stage of this study (second column), substantial changes were observed. Although the performances of C4.5 and k-NN improved marginally, FURIA generated poorer results. Nevertheless, SMO and NMEEFSD improved significantly. Indeed, the values with preprocessing for these algorithms are over and above the results obtained for any other algorithm used in the study. The creation of artificial NPC1 disease patients (performed by SLSMOTE in the training dataset) achieves an improvement in the test results, which are very relevant to this complex problem. Notably, the dataset is characterised by an imbalanced low number of samples with a high number of features. As can be observed in Table 2, the NMEEFSD algorithm yields the best result for this experimental study, with high-quality and competitive solutions in the scenario of predictive imbalanced datasets, although this algorithm was created from a descriptive perspective.

### 3.2. Supervised Descriptive Rules Obtained by NMEEFSD

Successful acquisition of the best results for the NMEEFSD algorithm encouraged us to make a careful analysis from the descriptive perspective, which is the nature of this algorithm. The main goal of this analysis is therefore to provide researchers with simple and interesting knowledge which could be employed in an easy manner in order to describe the problem.

- For describing the rules obtained for this algorithm, it is important to highlight the following:

The use of fuzzy logic allows us to represent variables with continuous domains as linguistic ones (values are represented through fuzzy linguistic labels in fuzzy sets [49]). These fuzzy sets are specified by means of uniform triangular partitions in order to facilitate the application to “real-world” problems because the representation of continuous variables is very close to human reasoning, e.g., a variable could be represented with three linguistic labels, making it possible to achieve an improved analysis. Specifically, for this study, we employed three linguistic labels for each variable, i.e., *low*, *normal* and *high*.

- Quality measures analysed for SD have a domain within the interval [0, 1], and these are relevant to measurements of the quality of the rules obtained with respect to trade-off between generality and precision, and interest. More information about these quality measures can be found in Appendix A.

### 3.3. Over-Representation (Enrichment) Analysis

For this investigation, a qualitative metabolite set enrichment analysis (ORA) was also performed. Notably, this form of Metabolite Set Enrichment Analysis (MSEA) directly investigates whether or not a series of functionally associated metabolites are represented or over-represented by selected metabolic pathways or routes without any requirement to preselect metabolites on the basis of a superficial, often subjective cutoff threshold value. Hence, it offers much promise for the identification of only slight, but nevertheless consistent, changes amongst a group of related biomolecules, which may indeed not be detected using more conventional arbitrary approaches. Here, ORA was implemented using the hypergeometric test to evaluate whether a particular metabolite set is represented more expectedly than by chance within the provided lists of compounds present in a range of metabolic pathways. One-tailed *p* values were provided after adjusting for multiple testing.

For this purpose, a software option containing 99 metabolite sets based on normal human metabolic pathways was used. Firstly, all biomolecular markers featured in both NPC1-disease-distinguishing rules R6 and R7 were featured; these subgroup biomarkers were xanthurenate and nicotinate (R6 alone), *p*(4)-aminobenzoate, hippurate, quinaldate and trigonelline (both R6 and R7), and indoxyl sulphate and 3-methylhistidine (R7 only). This analysis showed that the significant metabolic pathways involved in the NPC1 disorder were methylhistidine metabolism (enrichment ratio (ER) 85.5), β-alanine metabolism (ER 10.0), nicotinate and nicotinamide metabolism (ER 9.3), histidine metabolism (ER 7.9) and finally tryptophan metabolism (ER 5.7); raw *p* values for these were 0.0117, 0.0996, 0.105, 0.121 and 0.116, respectively; however, all corresponding false-discovery rate (FDR)-corrected *p* values were, as expected, >0.05).

**Figure 3:**
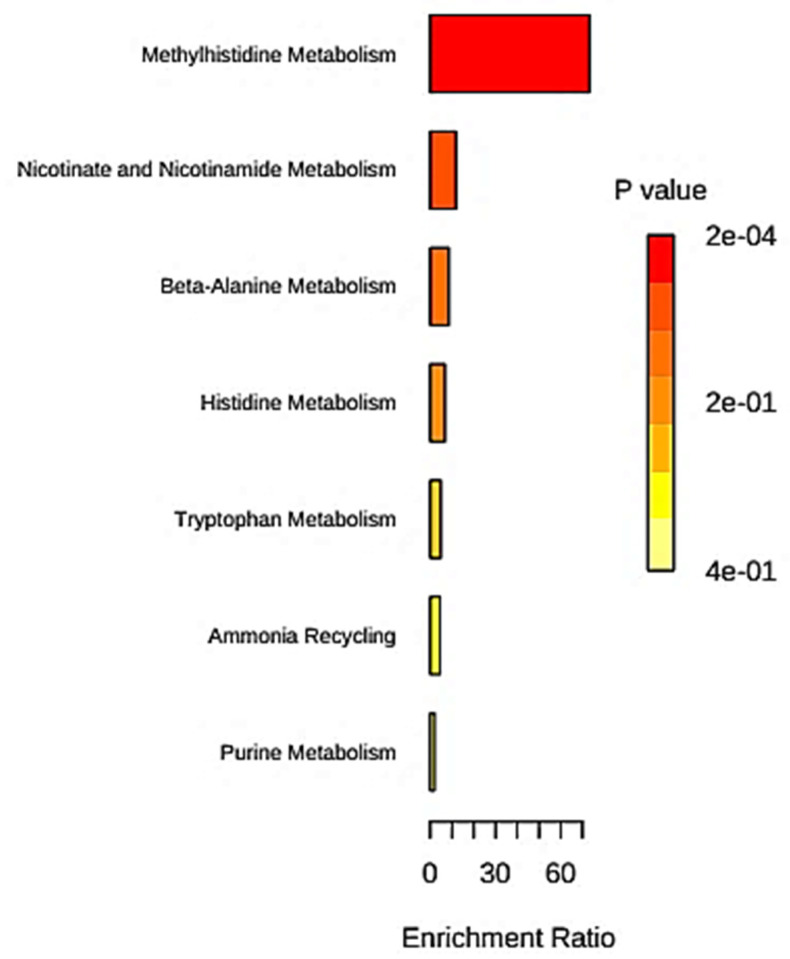
Over-Representation Enrichment Analysis of named metabolites implicated in all rules (R1–R7) as directive subgroup variables. Key metabolic pathways involved in the ^1^H NMR-based metabolomics diagnosis of NPC1 disease are shown, together with their enrichment ratios and raw *p* values for statistical significance. Abbreviations: 4 × 10^−1^, 2 × 10^−1^ and 2 × 10^−4^ represent *p* values of 4 × 10^−1^, 2 × 10^−1^ and 2 × 10^−4^ respectively.

Secondly, from Rule 1 to Rule 7, all metabolites deviating from their “normal” urinary concentration levels were included, and for this model the metabolic pathways featured were methylhistidine metabolism (ER 85.47), nicotinate and nicotinamide metabolism (ER 18.52), ammonia recycling (ER = 10.66), β-alanine metabolism (ER = 10.04), and histidine metabolism (ER = 7.94); raw *p* values for these were 0.0117, 0.00373, 0.0909, 0.0964 and 0.121, respectively (although all FDR-corrected values were again not significant). Finally, all metabolites implicated in all rules as directive subgroup variables were included in the enrichment analysis model, and this approach found that, again, methylhistidine metabolism was the most important (ER = 73.26), followed by the nicotinate and nicotinamide (ER = 11.86) > β-alanine (ER = 8.62) > histidine (ER = 6.82) > ammonia recycling (ER = 5.32) > tryptophan (ER = 4.88) > purine (ER = 1.98) metabolic routes (Figure 3). This final group of pathways was the only one that had FDR-corrected *p* values below or close to the significance level but in this case only for the methylhistidine (*p* = 0.0234) and nicotinate and nicotinamide (*p* = 0.0675) metabolic pathways.

However, it is important to note that such analysis is based on the conventional number of biomolecular “hits” on individual metabolic pathways and determinations of the statistical significance of these (Section 2.8), an approach which is clearly distinct from the SD algorithmic approach employed in this investigation.

Interestingly, the enrichment analysis performed also found that there were metabolic connectivities between the β-alanine and histidine pathways, with the latter then feeding into the nicotinate and nicotinamide and/or the ammonia recycling routes.

## 4. Discussion

An experimental study using data mining methodology in order to analyse a very complex problem, the CIT-based diagnosis of Niemann–Pick type C1 disease, is presented herein. To achieve this task, an ^1^H NMR-linked urinary dataset was collected, spectrally interpreted and analysed. This methodology is noninvasive, with a cost lower than that associated with other studies such as genomic-based ones. Once samples were prepared, the study from predictive and descriptive induction was carried out. The results of the study showed the importance of the application of intelligence techniques to “real-world” problems and, specifically, in medical domains where the combination between excellent infrastructure equipment and complex computational methodologies can throw new knowledge to researchers on rare cases such as the NPC1 disease.

This Discussion section is divided into two parts. Initially, a discussion regarding the SD viewpoint is performed, and then a qualitative over-representation metabolic pathway network enrichment analysis of the results acquired and their potential ramifications are presented in different subsections.

### 4.1. Analysis from Subgroup Discovery

As noted in previous sections, the discussion for SD must consider interpretability, interest and good trade-off between generality and precision. In this manner, we observed the following:

- All subgroups obtained have a low number of variables. For example, subgroups for the control heterozygous carrier class are between three and four variables, and for the NPC1 class, the subgroups have between six and seven variables. These values are low with respect to the whole dataset, which contains 54 continuous variables. This property shows the advantages of the use of this type of algorithm in order to analyse complex problems such as this one.
- The unusualness values are very interesting with values in the interval [0.55, 1.0]. As we have presented in ref. [50], we can indicate that all subgroups are contrasting and also serve as emerging rules. Specifically, it is interesting to note that values greater than 0.8 obtained for the subgroups of the NPC1 disease class show the unusualness and interest of the subgroups obtained.
- The relation between *TP_rate_* and fuzzy confidence is good. For the heterozygote class, this relation is excellent with values in confidence close to 1.0, along with excellent values in general. Nevertheless, in the NPC1 class, it should be noted that all or almost all examples of these collected samples are covered by the subgroups, respectively. However, despite the subgroups obtained in this class being specific, their confidence criterion values are somewhat lower.
- Finally, it should be considered that all values obtained for the *TEF p* value parameter are lower than the α = 0.10 considered in the experimental study, so all subgroups reject the null hypothesis, i.e., subgroups are interesting because there are significant differences between the proportions of positive and negative examples covered and not covered for each rule.

**Figure 4:**
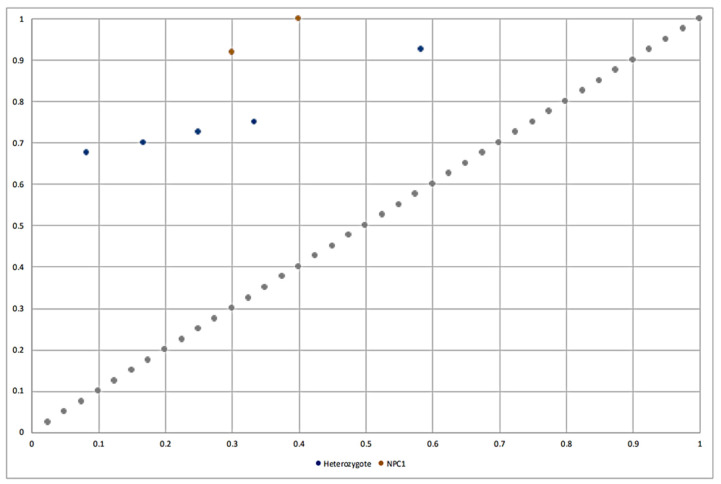
ROC space *TP_rate_*/*FP_rate_* for the subgroups obtained using the NMEEFSD algorithm.

The ROC analysis for the space *TP_rate_* and *FP_rate_* can be observed in Figure 4. This analysis was used in ref. [51] in order to discard those rules with a relation between *TP_rate_* and *FP_rate_* close to the main diagonal. This is primarily attributable to rules with the *TP_rate_*/*FP_rate_* ratio on the main diagonal having the same percentage distribution of covered positives and negatives (i.e., *TP_rate_* = *FP_rate_*) as the distribution in the entire dataset. In this manner, all subgroups obtained in the experimental study are valid and relevant from this point of view. Specifically, we can observe subgroups for the NPC1 class with the highest values in *TP_rate_* and good relationships with respect to the *FP_rate_* values.

It is very important to note the quality of the NMEEFSD algorithm in this type of complex problem in predicting new instances for the NPC1 disease class. As can be observed, in Table 2 the best results in AUC are obtained for this algorithm with the preprocessing SLSMOTE applied in a previous stage, i.e., NMEEFSD yields improved results compared to a support vector machine algorithm, and also the well-known C4.5 algorithm. However, as we have noted throughout the contribution, the NMEEFSD algorithm is not a classifier, it is a descriptive model. This fact is very relevant in view of the unusualness quality measure in the core of the algorithm. Indeed, the unusualness criterion measures the balance between the coverage and the gain in accuracy for the rule. These factors yield a potential difference to the rules obtained for the SD algorithms.

#### Potential Practical Applications of SGD, Including the Diagnosis and Prognosis of Diseases, and Therapeutic Interventions, Including Drug-Targeting Regimens 

The valuable diagnostically and/or prognostically trackable disease biomarkers found in the NMR-linked SD investigations performed here may indeed provide options for the design and development of novel, handheld biosensor devices which, at least in principle, can be operated and applied in a PC-controlled multi-analyte format, and which may achieve the detection and quantification of five or six of the most clinically significant biomarkers discovered. This strategy may provide an indispensable technology for the evaluation of NPC1 and other lysosomal storage diseases in an MV context, i.e., that involving rapid urine screening at patient points of contact (for example, at dedicated health clinics and services, clinical practitioner practices, etc.).

In addition to the provision of useful diagnostic and disease monitoring biomarker information to clinical and health service staff, the novel SD approaches developed here will potentially also inform researchers of modifications to intracellular metabolic pathways and functions in NPC1 and related diseases, including information regarding the availability of potential interventional sites for drug actions, notably those for therapeutic agents which are either already established or which are newly developing. Indeed, the identification, detection and validation of disease biomarkers, and their metabolic pathway impacts and longevities, are of critical importance for these considerations, as are the identification of cellular activities that are or may be affected by the upstream activation or downstream inhibition of disease-specific target proteins. Of course, also critical is the rich information potentially available on the actions of genes/proteins for drug-targeting technologies in diseases explored, together with the employment of fully validated biomarkers for evaluating dose-dependent drug actions and activities *in vivo* (distinguishing biomarkers may also have important roles as ‘hubs’ or ‘bottlenecks’ within these pathway systems).

However, it is of much pertinence to note that full validation of such biomarkers may only be accomplished when it is strongly statistically verified that they are able to respond positively to therapeutic avenues with pre-established, already-known and effective disease-modifying regimens for patients with the clinical condition considered. Additionally, it is also recommended that SD- and corresponding metabolomics analysis-identified biomarker strategies should be used only when fully validated, and when this is the case, they should be employed in parallel with other, more conventional or established methods for the clinical diagnosis of diseases, for example, histopathology gradings and supporting microscopic, haematological or microbiological evaluations, etc., where available. Indeed, this approach will permit researchers to explore significant associations between these two diagnostic routes of recognition. If such correlations are strong, these ^1^H NMR-linked technologies may serve to offer essential supporting diagnostic information.

With respect to the prognostic monitoring of disease progression and the potential successes of therapeutic agents applied to differential disease subclasses and stages, currently there is a high level of interest in treatment strategies that feature the prior stratification of patients according to their biological classifiers of disease (for example, those based on gene expression profiling), or alternatively the degree of their responses to selected drug therapies. One informative example of this is Hodgkin’s lymphoma (HL) [51,52]; indeed, despite the overall effectiveness of chemotherapeutic treatments for HL, ~15% of patients remain refractory to treatment, or alternatively will relapse. Hence, such patients may require more intensive chemotherapeutic treatments or the institution of newly developed, perhaps novel, therapeutic regimens [51]. Previously conducted UK-based clinical trials have explored the value of interim positron emission tomography and computed tomography (PET-CT) scanning to determine up- or downregulated treatment regimens, or other PET-CT scanning-based approaches (which serve to determine whether or not a radiotherapy option can be omitted for patient groups with early-stage disease), and these aim to successfully stratify patients into treatment-responsive or -unresponsive groups. Notwithstanding, since PET-CT scanning technologies are expensive and require expert interpretational skills, cheaper screening systems that are easier to implement and interpret may indeed serve to provide essential clues for patient stratification, and therefore the ^1^H NMR-based SD techniques presented here offer much value, most especially since this HL diagnostic problem again has an imbalanced experimental design. Hence, in this situation, patients presenting with an SD-determined poor prognostic potential may receive more intense treatments, whereas those with a more favourable prognostic status may be spared the harmful adverse side effects experienced with chemotherapeutic treatments.

Of much relevance to the clinical interventional prospectives of the SD techniques described in the current study, quite a large number of major developments in the molecularly targeted drug discovery research area have involved small-molecule anti-cancer drugs. Such advancements have resulted in an expanding number of successful treatments that have improved the prognostic outcomes of a range of cancer patients globally. Notable examples are (1) the therapeutic application of anti-oestrogens and anti-androgens as therapies for the treatment of hormone-driven breast and prostate cancers, (2) the advantageous curative therapeutic activity of all-*trans* retinoic acid for treating a high percentage of patients with acute promyelocytic leukaemia who bear translocations in the retinoic acid receptor (RAR) α gene [53], and also the Abelson tyrosine kinase (ABL) inhibitor imatinib, a novel drug which has clearly validated the design and therapeutic application of small molecules available for the treatment and prolonged survival prospects of patient populations with chronic myeloid leukaemia (where the BCR–ABL translocation drives the malignancy) [54,55].

Further low-molecular-mass drug molecules targeted on critical cancer targets include the epidermal growth factor receptor (EGFR) kinase inhibitors gefitinib and erlotinib for non-small-cell lung cancer (NSCLC) patients, and the vascular epidermal growth factor receptor (VEGFR) kinase inhibitor sorafenib for the treatment of renal cancer, along with the EGFR/ERBB2 inhibitor lapatinib for ERBB2-positive breast cancer [56]. Interestingly, the ‘oncogene addiction’ system, which is known as the ‘Achilles heel of cancer’, may also be viewed as a powerful rationale for the employment of molecularly targeted therapies for selected cancer conditions [57,58,59].

These examples clearly provide much support for the ‘targeting’ of metabolic profile and pathway perturbations, which indeed appear to play important roles as pathogenic ‘drivers’ in many human diseases.

### 4.2. Metabolic Disturbances in NPC1 Disease Indicated by Imbalances in the Urinary ^1^H NMR Profiles of Patients with This Disorder

#### 4.2.1. Tryptophan-Nicotinamide Metabolic Process

Tryptophan represents one of the 9 essential amino acids, and of critical importance to this study are linkages between tryptophan and nicotinamide metabolism, i.e., the combined tryptophan–nicotinamide metabolic process, which features no fewer than 8 out of 14 of the above metabolites featured in this discriminatory CIT SD model, and which is localized in the liver. This two-phase pathway biosynthesizes niacin (collectively known as nicotinamide and nicotinate, and which is viewed as a by-product of the kynurenine pathway) and is known to be critically influenced by physiological status, together with selected disease processes and the effects exerted by nutrients, hormones and xenobiotics [60]. In this joint pathway, tryptophan is metabolised to nicotinamide (and then immediately thereafter to 1-methylnicotinamide, also a key urinary biomarker variable identified herein), the first phase of which involves the 6-step conversion of this amino acid substrate to quinolinate sequentially, which features its prior transformation to α-amino-β-carboxymuconate-E-semialdehyde (ACMS). ACMS is then predominantly metabolically converted to α-aminomuconate-E-semialdehyde via the actions of ACMS decarboxylase, a reaction which yields picolinate, or alternatively glutamyl-CoA and, in turn, acetyl-CoA, on entry to the tricarboxylic acid cycle (TCA). However, some of this semialdehyde precursor cyclizes to quinolinate.

The second phase of this pathway, which involves the NAD cycle and nicotinamide catabolic routes, involves (1) transformation of quinolinate to nicotinate mononucleotide (catalysed by quinolinate phosphoribosyltransferase and featuring 5-phosphoribosyl-1-pyrophospahe and nicotinate reactants); (2) conversion of this product to NAD via nicotinate adenine dinucleotide; and (3) hydrolysis of NAD to nicotinamide. The latter then enters the NAD cycle and is catabolised to N-methyl-2-pyridone-5- and N-methyl-4-pyridone-3-carboxamides (2Py and 4Py, respectively) through its primary sequential conversion to 1-methylnicotinamide. Moreover, trigonelline is a metabolite of niacin, and quinaldate also represents a product of L-tryptophan catabolism and is generated from kynurenate.

However, since trigonelline is a caffeine metabolite, the significantly elevated ^1^H NMR-detectable concentrations of this agent found in the heterozygous (parental) control cohort of participants (*p* = 2.2 × 10^−4^, univariate two-sample *t* test performed on glog-transformed data, along with the SD rules developed, as shown in Table 3) may presumably arise from a much-increased consumption level of coffee and other caffeine-rich beverages in this adult group over that of the child-based NP-C1 disease group. This observation was also reported in a previous NMR-based metabolomics study [61], which featured some more conventional approaches to the MV statistical analysis of the datasets acquired. Results obtained concerning the different quality measures for each rule developed are shown in Table 4.

Further important considerations are that (1) tryptophan serves as a critical precursor of the neurotransmitters serotonin and melatonin; (2) tryptamine-derived quinolinate potentially exerts a role in neurodegenerative conditions, for example, it may act as a brain excitotoxin when discharged from activated macrophages [62]; (3) nicotinate’s derivatives NADH, NAD, NAD^+^ and NADP have roles as fundamental mediators of many biological processes, including energy metabolism, mitochondrial functions, calcium homeostasis, oxidative stress, gene expression, immunological functions, ageing and cell death [63]; (4) diminished urinary excretion of 1-methylnicotinamide provides evidence for niacin deficiency, and elevated urinary levels are observed in patients with severe liver damage (cirrhosis), this enhanced methylation status affording protection against any toxic effects exerted by rising intracellular nicotinamide accumulation; and (5) as noted above, the niacin metabolite trigonelline is present in coffee and many other plant sources and serves as a biomarker for the human ingestion of this common beverage, along with further trigonelline-rich dietary sources such as soy products and legumes.

#### 4.2.2. Kynurenine Pathway

Tryptophan is also metabolized via the kynurenine pathway, which is frequently systematically upregulated on activation of the immune response. The clinical significance of imbalances in this pathway are firstly coupled up- and downregulations in kynurenines and tryptophan, respectively, which exert a major regulatory impact on the immune response. Secondly, kynurenate, 3-hydroxykynurenate and quinolinate are neuroactive [64]. Therefore, defects in this pathway have been implicated in the pathogenesis of a wide range of human diseases, especially neurological disorders.

Intriguingly, higher [kynurenine]:[tryptophan] concentration ratios, and corresponding depleted tryptophan levels in blood serum, cerebrospinal fluid and/or brain tissue, are actually substantial in neurodegenerative diseases over and above that observed in a normally ageing population [65,66]; many of the studies performed to explore this involved age-matched healthy control participants. Pathologies linked to downstream upregulations in this kynurenine route include a broad spectrum of neurological conditions (e.g., Alzheimer’s and Huntington’s diseases, and amyotrophic lateral sclerosis), affection disorders (depression, anxiety and schizophrenia), autoimmune diseases such as multiple sclerosis and rheumatoid arthritis, peripheral disorders (for example, cardiovascular diseases), malignancy (colorectal cancer and haematological neoplasia) and various infectious diseases, e.g., HIV.

However, from these considerations, particularly important is the involvement of neurotoxins generated in this critical pathway and also alterations in the levels of neurotransmitters such as melatonin and serotonin. Indeed, selected [metabolite]:[tryptophan] concentration ratios can provide much valuable information regarding the degree of neuroinflammation featured in neurodegenerative diseases [67].

Also notable as key biomarker variables in the current study are further metabolites involved in the tryptophan metabolic pathway, specifically xanthurenate and indoxyl sulfate. The former of these, a quinolone carboxylate derivative, forms part of the pathway subroute from 3-hydroxy-L-kynurenine, its immediate precursor being 4-(2-aminophenyl)-2,4-dioxobutanoate, whereas the latter is the sulphated urinary excretion product of indoxyl (indoxyl sulphate), which arises from tryptophan itself via indole.

#### 4.2.3. Imbalances in Tryptophan Metabolism: Relevance to Lysosomal Storage Diseases

Disturbances in L-tryptophan metabolism have been considered as key features associated with fibromyalgia syndrome, a comorbidity experienced by some patients with symptomatic Gaucher’s disease [68], which is another, albeit the most common, lysosomal storage disease. Indeed, Schwarz et al. [69] investigated altered tryptophan metabolism, along with the depletion of this amino acid, in *n* = 17 fibromyalgia patients; although these cases reacted similarly to an age-matched healthy control group for most markers evaluated, the fibromyalgia patient cohort had significantly elevated interleukin-6 (IL-6) concentrations. Such results indicated modifications in tryptophan metabolism, since diminishing tryptamine levels activate both 5-hydroxytryptamine (serotonin) metabolism and IL-6 generation. However, in healthy control subjects, a tryptophan depletion-mediated experimental and transient interruption in global monoamine species function was not found to induce IL-6 production [70]. In Gaucher’s disease patients, however, IL-6 concentrations were increased significantly [71]. Additionally, it appears that an IL-6 174G→Cpromoter polymorphism (of GIC genotype) is linked to a milder Gaucher’s disease phenotype, and therefore, it could play a mitigating genetic-modifying role [72].

Since the kynurenine pathway serves as the primary route for tryptophan catabolism in the liver, and also the preliminary point for nicotinamide adenine dinucleotide biosynthesis in mammals, imbalances involving either its dysregulation or overactivation may give rise to immune system activation and the adverse bioaccumulation of potential neurotoxins. Such phenomena render this pathway a valuable target for therapeutic intervention in order to treat inflammation and selected neurological diseases, most particularly in cancer patients receiving chemotherapeutic regimens [73]. Such drug targeting has largely been focused on the major rate-limiting enzymes indoleamine-2,3-dioxygenase 1 (IDO1), IDO2 tryptophan-2,3-dioxygenase (TDO) and kynurenine monooxygenase (KMO) [74].

#### 4.2.4. 3-Hydroxyphenylacetate and Tyrosine Metabolism

3-Hydroxyphenylacetate is involved in the human tyrosine metabolism pathway, in which it is transformed to 3,4-dihydroxyphenylacetate via the enzyme 4-hydroxyphenylacetate 3-mono-oxygenase, a reaction which also requires the NAD^+^/NADH coenzyme system. Perturbations to biofluid and tissue concentrations of 3-hydroxyphenylacetate have been correlated to inborn errors of metabolism and phenylketonuria [75]. Moreover, previous ^1^H NMR investigations of the pathogenesis of developing NPC1 disease-associated hepatic dysfunction in a selected mouse model have revealed that hepatic 3-hydroxyphenylacetate levels were significantly lower in this classification than those of heterozygous controls (NAD cycle biomarkers also contributed towards this distinction) [76]. Such perturbations to 3-hydroxyphenylacetate biosynthesis in NPC1-diseased mice suggested perturbations to the hepatic [NAD^+^]:[NADH] molar concentration ratio, which represents an index of redox status within mitochondria. Although not featured as a key discriminatory subgroup metabolite in the human dataset explored in the current study, its creatinine-normalised urinary concentration was significantly lower in a univariate context (*p* = 0.049, rigorous Welch and Brown–Forsyth F ratio statistics).

However, the potential applications of 3-hydroxyphenylacetate as a biomarker for NPC1 disease remain complex and unclear, since this phenolic acid anion is also generated as a co-metabolite arising from gut microflora [77]. A further complication is that 3-hydroxyphenylacetate may also arise directly from dietary sources [78], and it can be generated from dietary polyphenolic precursors.

Although previous reports available on the value of 3-hydroxyphenylacetate as a biomarker are somewhat limited, Boudonck et al. [79] found that, along with hippurate, this metabolite represented a progressive downregulated urinary biomarker of nephrotoxicity in rats receiving three different nephrotoxins. Notably, hippurate was also found to serve as a key biomarker in our previously reported NP-C1 disease-associated liver damage dataset [76].

#### 4.2.5. Significance of Further Non-Tryptophan-Nicotinamide/Tryptophan-Kynurenine Pathway Metabolites Featured in the SD Models Developed

Of the other non-tryptophan–nicotinamide pathway metabolites featured in the SD models developed here, histidine represents an essential precursor for the biosynthesis of both histamine and carnosine and interestingly is involved in a variety of mental and physical retardation problems, for example, diminished intellectual function, ataxia, tremor and psychoses. However, 3-methylhistidine arises from the methylation of actin and myosin, and peptide bond biosynthesis, and serves as a biomarker for muscle protein degradation [80], and therefore, it may be of relevance to the multifarious pathogenic and chemopathological cascades associated with NPC1 disease.

Moreover, the purine derivative hypoxanthine is an adenosine intermediate involved in the salvage pathway of nucleic acid production, whereas urinary concentrations of hippurate, an excretion product derived from the conjugation of glycine with benzoate, are elevated with increasing levels of dietary phenolic compound consumption [80].

Our previously conducted metabolomics investigation discovered a wide range of urinary metabolites in patients with NPC1 disease which were significantly modified in concentration when compared to a larger cohort of parental heterozygotic carriers of this disease [61], although that study also included selected candidate aliphatic biomolecules, the datasets from which were analysed by a wealth of standard metabolomics techniques, and correlated component regression (CCR) analysis, along with CIT approaches, the latter including genetic algorithm techniques. Indeed, this previous study found that the low-field ^1^H NMR region of (aromatic) metabolites which qualified as significant biomarkers were upregulated urinary quinolinate and 3-methylhistidine and downregulated nicotinate, 1-methyl nicotinamide, N-methyl-2-pyridone- and N-methyl-4-pyridone-5-carboxamides (2PY and 4PY, respectively), and the caffeine metabolite trigonelline, although the latter may have arisen from a much-diminished rate of caffeinated beverage consumption in the largely younger or much younger NPC1 disease group evaluated. Together with these aromatic biomarkers, a range of aliphatic ones were also detected, e.g., bile acids, 3-aminoisobutyrate and other branched-chain amino acid (BCAA) intermediates and degradation products, N-acetyl sugars, glutamine, creatine, succinate and trimethylamine, which were all NPC1 disease-upregulated. Extension of these datasets to quantitative metabolite set enrichment and pathway topological analyses revealed that bile acid biosynthesis, BCAA degradation, methylamine metabolism, pyrimidine (thymine) catabolism and the nicotinate and nicotinamide metabolism pathways were imbalanced in NPC1 disease and that the brain, liver, mitochondria, endoplasmic reticulum, and of course the lysosome, represented key tissue, organ and subcellular sites for important disease activities. However, for the current investigation, a qualitative metabolite set enrichment analysis performed revealed that the significant metabolic pathways involved were the methylhistidine, β-alanine, ammonia recycling, nicotinate and nicotinamide, histidine, tryptophan and purine metabolism routes, with methylhistidine metabolism being the most important (false discovery rate (FDR)-corrected *p* value 0.0234).

Hence, reassuringly, the pattern of key aromatic biomarkers found in the current study has many similarities to that found in our previous investigation reported in 2014 [61], with 3-methylhistidine, quinolinate, nicotinate, 1-methylnicotinamide and trigonelline being featured as key discriminating determinants. Also of importance is the involvement of ’normal’ (intermediary) urinary levels of *p*(4)-aminobenzoate as a critical feature in rules 6 and 7 for the diagnosis of NPC1 disease (Table 3). Although an important requirement for some bacteria, it appears that this biomolecule is not essential for humans, who lack the enzymes to transform it into folate. However, it is widely distributed in nature and therefore is present in many human and animal foods and tissues. It may also be biosynthesised by selected bacteria present in the human intestinal tract, e.g., *E. coli*, so it could be viewed as a co-metabolite in humans [80]. In rabbits, 4-aminobenzoate is predominantly metabolized to 4-acetamidobenzoate, 4-aminohippurate and 4-acetamidohippurate via acetylation and glycine conjugation pathways [81].

## 5. Conclusions

In conclusion, the novel SD techniques applied in this investigation for the first time provided much valuable knowledge regarding biomolecular and metabolic pathway imbalances potentially featured in the pathogenesis of NPC1 disease. This information was derived from an examination of disturbed, NPC1-mediated patterns of urinary metabolites exposed by high-resolution ^1^H NMR analysis. These perturbed metabolic pathways predominantly involved those comprising aromatic biomolecules, including amino acids, and these included (1) the joint tryptophan–nicotinamide metabolic system, which is known to be affected by physiological homeostasis and selected human disorders, with the essential amino acid tryptophan acting as a neurotransmitter precursor, and quinolinate playing an important role in neurodegenerative diseases in general; (2) the kynurenine pathway, which is efficiently upregulated by immune response activations; (3) tryptophan metabolism, which is of much relevance to the pathogenesis of lysosomal storage diseases; and (4) tyrosine metabolism, which features the metabolite 3-hydroxyphenylacetate, and which has previously been observed to be a potential biomarker for NPC1 disease-driven hepatic dysfunction in a mouse model, although it should be noted that it may also arise from dietary sources. Further metabolic routes implicated are those involving histidine and 3-methylhistidine, and the adenosine intermediate hypoxanthine, along with 4-aminobenzoate and the excretion product hippurate. The novel SD strategy employed for the analysis of MV data from this NPC1 study should be readily translatable to the analysis of other metabolomics datasets with only clinically limited sample sizes. 

## Figures and Tables

**Table 1:** Confusion matrix for a two-class problem.

Class/Prediction	Positive	Negative
Positive	True positive (TP)	False negative (FN)
Negative	False positive (FP)	True negative (TN)

**Table 2:** Detailed AUROC value results table for the algorithms used in the study. Only test results are shown for the original and the preprocessed dataset (SLSMOTE).

Algorithm	Original	SLSMOTE
C4.5	**0.6583**	0.6833
FURIA	0.5791	0.5458
k-NN	0.5750	0.5958
SMO	0.5833	0.7041
NMEEFSD	0.5500	**0.7125**

**Table 3:** Rules obtained by NMEEFSD for the whole dataset.

Rule	Description
R_1_	IF Hippurate-C3/5-CH=normal AND Histidine-C2-CH=low AND Hypoxanthine-C3/5-CH=normal AND Quinolinate-C5-CH=normal THEN Heterozygote
R_2_	IF Hippurate-C3/5-CH=normal AND Histidine-C2-CH=low AND Hypoxanthine-C3/5-CH=normal AND 1-Methylnicotinamide-C5-CH=low THEN Heterozygote
R_3_	IF p-Aminobenzoate-C3/5-CH=low AND Hypoxanthine-C3/5-CH=normal AND Quinolinate-C5-CH=normal THEN Heterozygote
R_4_	IF Hippurate-C3/5-CH=normal AND Hypoxanthine-C3/5-CH=normal AND 1-Methylnicotinamide-C5-CH=low THEN Heterozygote
R_5_	IF Hippurate-C3/5-CH=normal AND *p*-Aminobenzoate-C3/5-CH=low AND Hypoxanthine-C3/5-CH=normal AND Quinolinate-C5-CH=normal THEN Heterozygote
R_6_	IF Xanthurenate-C3-CH (s)=normal AND p-Aminobenzoate-C2/6-CH=normal AND *p*-Aminobenzoate-C3/5-CH=normal AND Hippurate-C2/6-CH=normal AND Quinaldate-C4-CH=normal AND Nicotinate-C2-CH=low AND Trigonelline-C2-CH=low THEN NPC1
R_7_	IF p-Aminobenzoate-C2/6-CH=normal AND Indoxylsulphate-C2/Phe-C2/6-CH=normal AND Hippurate-C2/6-CH=normal AND 3-Methylhistidine-C2-CH=normal AND Quinaldate-C4-CH=normal AND Trigonelline-C2-CH=low THEN NPC1

**Table 4:** Results of the different quality measures for each rule: Unusualness (Unus), True-Positive Rate (TP_rate_), False-Positive Rate (FP_rate_), Fuzzy Confidence (FCnf) and Fisher exact test (TEF) *p* value.

Class	Rule	Vars	Unus	TPrate	FPrate	FCn f	TEF
	*R*_1_	4	0.671	0.925	0.583	0.915	0.011
	*R*_2_	4	0.796	0.675	0.083	0.967	0.000
Heterozygote	*R*_3_	3	0.708	0.750	0.333	0.971	0.014
	*R*_4_	3	0.767	0.700	0.167	0.960	0.001
	*R*_5_	4	0.737	0.725	0.250	0.971	0.005
NPC1	*R*_6_	7	0.800	1.000	0.400	0.403	0.000
	*R*_7_	6	0.808	0.917	0.300	0.484	0.000

## Data Availability

The data presented in this study are available in this article.

## Appendix A. Quality Measures in Subgroup Discovery

The representation of the knowledge in SD tasks is performed through rules, and specifically, the representation using fuzzy logic is performed through fuzzy rules. It is necessary to present fuzzy subgroups in this section in order to understand their analysis. In Equation (A1), the representation of a canonical fuzzy rule for SD can be defined as:

(A1) $$R:IF\;X_{1}=\left({LL}_{1}^{2}\right)\;AND\;X_{3}=\left({LL}_{3}^{1}\right)\;THEN\;{Target}_{value}$$

where:

- *X* = {*X_m_*/*m* = 1, …, *n_v_*} is a set of features used to describe the subgroups, and *n_v_* is the number of descriptive features, e.g., a problem with *n_v_* = 3 such as *Age*, *Sex* and *Visits*.
- $LL_{n_{v}}^{l_{n_{v}}}$ is the *LL* number $l_{n_{v}}$ of the variable *n_v_*, e.g., a representation of three linguistic labels for the *Visits* variable (*V*) where $LL_{V}^{1}$ = *Low*, $LL_{V}^{2}$ = *Medium* and $LL_{V}^{3}$ = *High*
  In this manner, the quality measures analysed for this type of subgroups are:
- *Unusualness* is the weighted relative accuracy of a rule [82] which measures interest and a trade-off between generality and precision [50]. It can be computed as:

(A2) $$Unus\left(R_{i}\right)=\frac{TP+FP}{n_{s}}\left(\frac{TP}{TP+FP}-\frac{Positive}{n_{s}}\right)$$

It is best described as the balance between the coverage of the rule and its accuracy gain, where *n_s_* is the number of total examples, and *Positive* depicts all the examples of the target variable.

The domain of this quality measure is specified for each problem because there is a direct dependence with respect to the target variable. Therefore, it is necessary to normalise the value to the interval [0, 1] in order to facilitate the analysis. This normalisation can be analysed, as shown in ref. [50].

- *TP_rate_* is the proportion of actual matches that have been classified correctly [83], and it has a component based on generality. It is computed as:

(A3)
$${TP}_{rate}\left(R_{i}\right)=\frac{TP}{Positive}$$

This quality measure can be found in the literature as the support based on the examples of the class, *Recall* or *Sensitivity*, and its domain is [0, 1].

- *FP_rate_* is the proportion of instances that have been classified incorrectly for the nonclass of the rule. Its domain is [0, 1], and it is computed as:

(A4)
$${FP}_{rate}\left(R_{i}\right)=\frac{FP}{Negative}$$

- Fuzzy confidence is an adaptation of the standard confidence measure for fuzzy rules [84]. This quality measure obtains the precision of one subgroup. It has a domain [0, 1], and it is defined as:

(A5)
$$FCnf\left(R_{i}\right)=\frac{TP}{TP+FP}$$

## Appendix B. Subgroup Discovery through Evolutionary Fuzzy Systems

A fuzzy system [26] augmented with a learning process based on evolutionary algorithms [25] is defined as an evolutionary fuzzy system, as documented in ref. [27]. In this definition, two concepts are presented: fuzzy systems and evolutionary algorithms. The former are usually considered in the form of fuzzy-rule-based systems (FRBSs), which are composed of “IF-THEN” rules, where both the antecedent and consequent phases can contain fuzzy logic statements. Fuzzy systems are based on fuzzy logic [26], which already allows us to consider uncertainty, and also to represent the continuous variables in a manner that is close to human reasoning. In this manner, interpretable fuzzy rules consider continuous variables as linguistic ones, where values are represented through fuzzy linguistic labels in fuzzy sets [49]. These fuzzy sets are specified by means of uniform triangular partitions in order to facilitate the application to real-world problems because the representation of such continuous variables is very intuitive, e.g., a variable such as height could be represented with three linguistic labels such as Small, Normal and Tall, making it possible to achieve an improved analysis. In addition, the use of rules in order to represent the knowledge permits the explanation of decisions, and we may also check fairness, privacy, robustness (small changes in the input do not lead to large changes in the description) and trust [85], which are considered as ethics guidelines for trustworthy artificial intelligence [86].

However, evolutionary algorithms are stochastic algorithms for optimising and searching. These algorithms were introduced by Holland [87]. Different computational models can be found within these types of algorithms, such as genetic algorithms [87,88], evolution strategies [89], evolutionary programming [90] and genetic programming [91], amongst others. The evolutionary algorithms imitate the principles of natural evolution to address optimisation and learning problems, and they are well suited to perform the SD task in view of their ability to reflect the interaction of variables in a rule-learning process, which also provides much flexibility in the representation [92].

SD is a rule-learning process that can be viewed as an approximation problem in which the objective is the learning of the parameters of the model. In this task, the search space can be very complex, and the search strategy used becomes a key factor. The use of evolutionary fuzzy systems is very well suited to this task because:

- These algorithms perform a global search in space in a suitable mode, as can be observed in the ‘real-world’ problems solved in the literature, for example, in bioinformatics [21,93], medicine [22], E-commerce [94] or industry [95], amongst others.

Evolutionary fuzzy systems for subgroup discovery allow us to obtain knowledge very close to human reasoning, with descriptive rules containing linguistic labels on numerical variables, so that researchers may analyse such knowledge in a simple and intuitive manner.
